# Functions of Vγ4 T Cells and Dendritic Epidermal T Cells on Skin Wound Healing

**DOI:** 10.3389/fimmu.2018.01099

**Published:** 2018-06-04

**Authors:** Yashu Li, Jun Wu, Gaoxing Luo, Weifeng He

**Affiliations:** ^1^State Key Laboratory of Trauma, Burn and Combined Injury, Institute of Burn Research, Southwest Hospital, Third Military Medical University (Army Medical University), Chongqing, China; ^2^Chongqing Key Laboratory for Disease Proteomics, Chongqing, China; ^3^Department of Burns, The First Affiliated Hospital, Sun Yat-Sen University, Guangzhou, China

**Keywords:** wound healing, dendritic epidermal T cell, Vgamma 4 T cell, IL-17A, IGF-1, re-epithelialization

## Abstract

Wound healing is a complex and dynamic process that progresses through the distinct phases of hemostasis, inflammation, proliferation, and remodeling. Both inflammation and re-epithelialization, in which skin γδ T cells are heavily involved, are required for efficient skin wound healing. Dendritic epidermal T cells (DETCs), which reside in murine epidermis, are activated to secrete epidermal cell growth factors, such as IGF-1 and KGF-1/2, to promote re-epithelialization after skin injury. Epidermal IL-15 is not only required for DETC homeostasis in the intact epidermis but it also facilitates the activation and IGF-1 production of DETC after skin injury. Further, the epidermal expression of IL-15 and IGF-1 constitutes a feedback regulatory loop to promote wound repair. Dermis-resident Vγ4 T cells infiltrate into the epidermis at the wound edges through the CCR6-CCL20 pathway after skin injury and provide a major source of IL-17A, which enhances the production of IL-1β and IL-23 in the epidermis to form a positive feedback loop for the initiation and amplification of local inflammation at the early stages of wound healing. IL-1β and IL-23 suppress the production of IGF-1 by DETCs and, therefore, impede wound healing. A functional loop may exist among Vγ4 T cells, epidermal cells, and DETCs to regulate wound repair.

## Skin γδ T Cells are Heavily Involved in the Wound Healing Process

Wound healing is a complicated repair process to recover the integrity of skin. This process is orchestrated by four overlapping phases, which are clotting, inflammation, re-epithelialization, and remolding (1). Murine γδ T cells as important components of skin immunity engage in inflammation and re-epithelialization in wound repair (2–4). Several subsets of γδ T cells with distinct functions exist in skin tissue: dendritic epidermal T cells (DETCs), which uniformly express an invariant Vγ5Vδ1 TCR (according to Heilig and Tonegawa’s nomenclature) and exclusively reside in the murine epidermis (>90%), primarily provide IGF-1 and KGF-1/2 in the epidermis to enhance re-epithelialization and thereby promote skin wound repair. Vγ4 T cells, a dominant subset of murine peripheral and dermal γδ T cells (approximately 50%), provide an early major source of IL-17A to initiate and amplify local inflammation after skin damage (5, 6). Interestingly, although inflammation is required for efficient skin wound healing, excessive inflammation has a negative impact on skin wound repair. In line with this notion, IL-17A, a potent pro-inflammation factor, exhibits dual roles in skin wound closure (7–9). It has been reported that dermal Vγ4 T cells infiltrate the epidermis and interact with epidermal cells to form an IL-17A-IL-1/23 positive feedback loop for amplifying local inflammation. Li et al. recently revealed that Vγ4 T cells suppress IGF-1 production by DETCs through the IL-17A-IL-1/23 loop and thus delay skin wound healing (10). This suggests that a potential functional link exists between Vγ4 T cells, epidermal cells, and DETCs in the wound-healing process. This review focuses on the functional diversity of skin-resident γδ T cell subsets in wound repair.

## The Development of Vγ5 T Cells in the Thymus

Vγ5 T cells are the first generated γδ T cells on embryonic day (ED) 13 in the early fetal thymus, but they are no longer produced after ED 18 (11, 12). Their γ and δ chains are identically rearranged to Vγ5-Jγ1Cγ1 and Vδ1-Dδ2-Jδ2Cδ, respectively, with invariant canonical junctional sequences (13). IL-7 signaling is required for the rearrangement of TCRγ but not TCRαβ (14). IL-7 and IL-7 receptors are responsible for the recombination of the TCRγ locus by regulating locus accessibility to the V(D)J recombinase (15). Positive selection is necessary for the maturation of Vγ5 T cells in the fetal thymus, which depends on the engagement of TCR and some ligands expressed by thymic stromal cells (16). Skint-1 is essential for the positive selection of Vγ5 T cells in the murine fetal thymus (17). CD122 (the β chain of IL-2/IL-15 receptor, IL-2/IL-15Rβ) and the skin-homing receptors are induced on Vγ5 T cells after positive selection in the fetal thymus, and are crucial for Vγ5 T cells to migrate into the epidermis (16). Vγ5 T cells gain a “memory-like” pre-activation phenotype of CD44^+^CD122^+^CD25^−^ before exiting the thymus (11).

## The Migration and Residency of Vγ5 T Cells in the Epidermis

During ED 15.5–16.5, Vγ5 T cells egress from the thymus and move to the epidermal layer of skin (18). The expression of CCR6 is reduced, whereas the expression of sphingosine-1-phosphate receptor 1 (S1P1) is increased on mature Vγ5 T cells, both of which allow mature Vγ5 T cells to exit but retrain immature cells in the thymus (16). Furthermore, the expressions of skin-homing molecules CCR10, CCR4, E, and P selectin ligands, and integrin α_E_ are also markedly increased on the surface of Vγ5 T cells to help them migrate and reside in the epidermis (16, 19, 20). CCR9, CCR7, and CD62L have low expression on Vγ5 T cells, indicating that Vγ5 T cells are not able to migrate into secondary lymphoid organs (21).

Vγ5 T cells exclusively reside in the murine epidermis and comprise over 90% of murine epidermal T lymphocytes (22). Since the characteristic feature of epidermal Vγ5 T cells is their highly dynamic dendritic morphology, they are named DETCs. DETCs are anchored in the upper epidermis, and most dendrites are immobilized and apical toward keratinocyte tight junctions, while the remaining dendrites are projected to the basal epidermis and extend and contract in a highly mobile state (23). Keratinocytes predominantly express E-cadherin and DETCs express E-cadherin receptor integrin α_E_β_7_, especially at the ends of apical dendrites, which assist in anchoring the dendrites of DETCs in the epidermis (23).

## Homeostasis of DETCs in the Epidermis

Dendritic epidermal T cells slowly expand under a steady state to maintain skin homeostasis (23). Vγ5 TCR and Skint-1 signaling are required for DETC homeostasis in the epidermis (17). Several secreted factors also contribute to the maintenance of DETCs. For example, CD122 expressed on DETCs is essential for their proliferation and survival in both fetal thymus and skin (24). IL-15Rα (CD215) is highly expressed on the surface of DETCs (11). IL-15 helps the survival and proliferation of DETCs upon TCR engagement (25). Furthermore, IL-15 can interact with CD122 of DETCs to maintain their localization and homeostasis in the epidermis (25). Importantly, DETCs secrete a small amount of IGF-1 in a steady state to sustain survival and prevent apoptosis of keratinocytes to maintain epidermis homeostasis (4). Liu et al. and Bai et al. recently reported that DETC-derived IGF-1 is positively correlated with keratinocyte-derived IL-15, which is partially controlled by the mTOR signaling pathway (26, 27). In addition, epidermal IGF-1 and IL-15 cooperate to promote the homeostasis of DETCs in diabetic animals (28). CD122 and CD69 are regarded as activation markers on DETCs (23). Vγ5 TCR signals preserve the expression of CD69 and CD122, which help DETCs stay in a state of pre-activation (23).

## γδ T Cells in the Human Epidermis

Murine DETCs lack an exact counterpart in humans. γδ T cells have a TCR that expresses the Vδ1 chain reside in both the epidermis and dermis of human skin (29). γδ T cells that exist in peripheral blood express the Vδ2 TCR (30). Cutaneous leukocyte antigen (CLA), which is the ligand for E-selectin and a skin-homing marker, is also expressed on epidermal- and dermal-resident Vδ1 T cells and αβ T cells, while CLA expression on Vδ2 T cells from the blood is low (29). No significant or distinct differences in CLA expression on Vδ1 T cells or αβ T cells exist between the epidermis and dermis. Vδ1 TCR comprise about 10–20% of T cells in the human epidermis and dermis, respectively, while the ratio of Vδ1 T/αβ T cells is less in the epidermis than in the dermis. Epidermal-resident Vδ1 T cells and αβ T cells are activated after acute injury and produce IGF-1 to promote wound repair. However, both Vδ1 T cells and αβ T cells separated from chronic non-healing wounds do not secrete IGF-1, indicating that their function is impaired in chronic wounds compared with T cells isolated from acute wounds (29). Moreover, human blood Vδ2 T cells can be recruited to the skin inflamed with psoriasis. These Vδ2T cells are CLA- and CCR6-positive, secrete proinflammatory cytokines, such as IL-17A, TNF-α, and IFN-γ, and produce psoriasis chemokines, such as IL-8, CCL3, CCL4, and CCL5 (31). CLA^+^Vδ2 T cells not only upregulate the production of IGF-1, but also activate keratinocytes dependent on TNF-α and IFN-γ (31). Skint-1 is absent in humans, which may partially explain why the development of TCR chains is different between humans and mice (17).

## The Activation of DETCs upon Skin Injury

The activation of DETCs around wound edges is necessary for their proliferation and the secretion of epidermal growth factor during wound healing (3, 4, 32). Once keratinocytes get stressed or damaged, the morphology of DETCs changes from dendritic to round at the wound edge 1 h later (3). TCR complexes locate at the apical dendrite ends under a steady state, but migrate to the basal epidermis upon wounding (23). Functional changes follow the morphological changes, and the expression of IGF-1 is markedly increased in DETCs (4). Differing from αβ T cells, γδ T cells are directly activated by TCR signaling in a non-major histocompatibility complex (MHC)-restricted manner (33). TCRs on DETCs sense some unidentified ligands expressed on damaged keratinocytes after skin injury (34). Apart from TCR signaling, other co-stimulatory molecules [such as NKG2D, junctional adhesion molecule-like protein (JAML), and 2B4] and T cell growth factors have been recently demonstrated to contribute to the activation of DETCs (35–38).

## NKG2D

NKG2D is a C-type lectin-like stimulatory receptor, expressed on activated CD8^+^T cells, macrophages, NK1.1^+^T cells, and DETCs (39). NKG2D has two alternative splicing isoforms: NKG2D-S (short) and NKG2D-L (long) (40). DETCs constitutively express NKG2D-S, NKG2D-L, and cell surface protein NKG2D (40). NKG2D signals act as co-stimulatory signals for CD8^+^T cells, or directly trigger cytotoxicity and cytokine production in activated murine NK cells (39). Without TCR engagement, NKG2D signals are sufficient to trigger cytotoxicity and IFN-γ production in DETCs (40). NKG2D ligands, which belong to MHC-class-I related proteins, are expressed under stress conditions, such as infection, tumorigenesis, and tissue damage (41). Retinoic acid early inducible-1 α-ε, mouse UL16-binding protein-like transcript 1, and histocompatibility a-c (H60 a-c) are known NKG2D ligands in mice, and among them H60c has been detected in skin (42). H60c protein is inductively expressed on keratinocytes at wound margins (43). The interaction between NKG2D and H60c is necessary for KGF secretion by DETCs in wound repair (43).

## Junctional Adhesion Molecule-Like Protein (JAML)

Junctional adhesion molecule-like protein is expressed on the surface of DETCs (44). Coxsackie and adenovirus receptor (CAR) is induced on damaged keratinocytes and acts as a functional ligand for JAML (44). The JAML–CAR interaction is necessary for the activation of DETCs and cytokine production of TNF-α and KGF-1 (44). Blocking the interaction between JAML and CAR impedes wound healing, suggesting that JAML–CAR provides a costimulatory signal for the activation of DETCs during wound repair (44).

## 2B4

2B4, a 66-kD glycoprotein, is expressed on NK and T cells and kills tumor targets by non-MHC-restricted mechanisms (45). 2B4 is also detected on DETCs and helps DETCs to mediate cytotoxic killing against skin-derived tumors (46). IL-2 upregulates 2B4 expression and enhances cytotoxic ability of DETCs (46). Whether 2B4 participates in wound healing has not been clarified.

## Toll-Like Receptor (TLR) 4

Toll-like receptor 4 is the primary signaling receptor for lipopolysaccharide (LPS) (47). MD2 assists TLR4 for intracellular distribution and accelerates TLR4 for LPS recognition (48). In the steady state, the expression of TLR4-MD2 is lacking on the surface of DETCs, while during cutaneous inflammation, TLR4-MD2 expression is improved on DETCs when they migrate from the epidermis (47). The roles of TLR4-MD2 in wound healing are not known.

## IL-15

**Figure 1:**
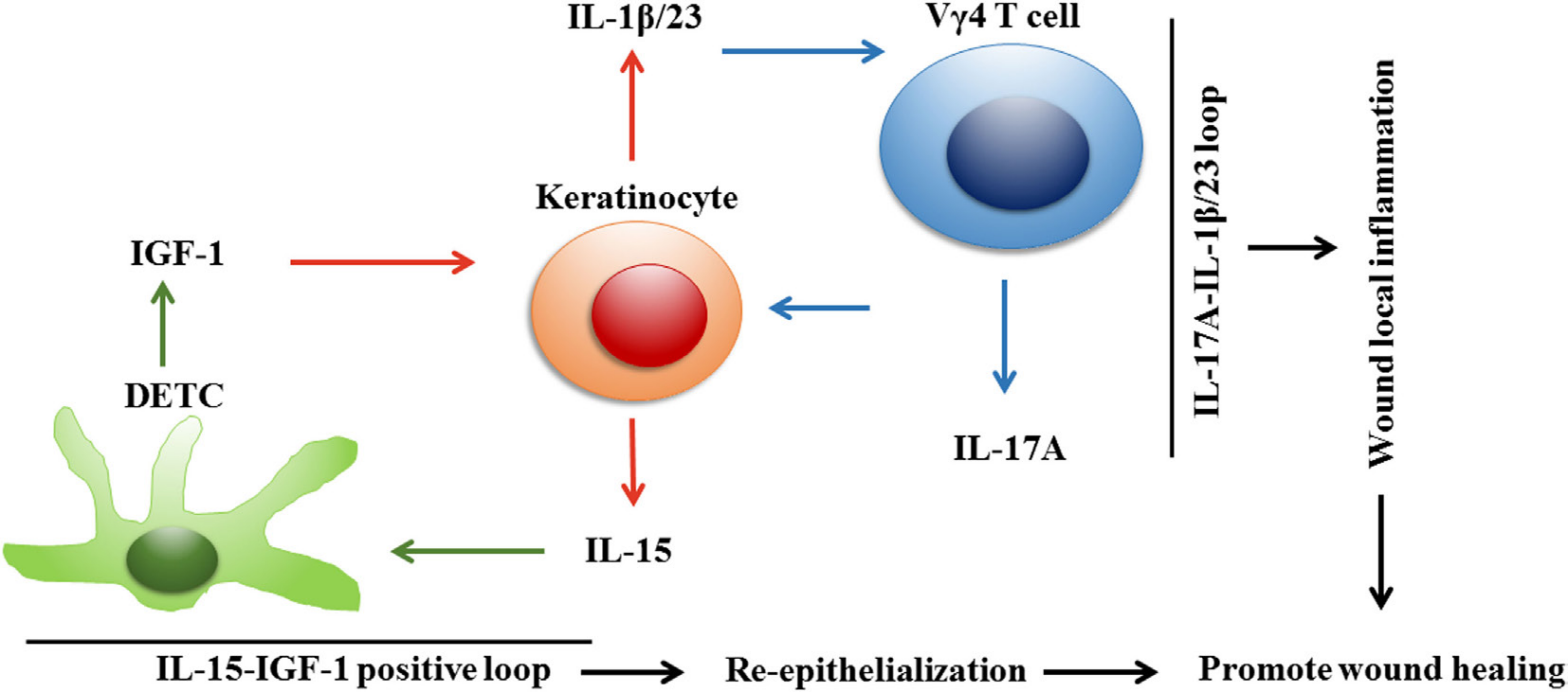
Dendritic epidermal T cells (DETCs), keratinocytes, and Vγ4 T cells constitute two correlated loops to improve wound repair in diabetic mice. Upon skin injury in diabetic mice, keratinocyte-derived IL-15 increases IGF-1 production by DETCs, which in turn enhances keratinocytes to secrete IL-15. A positive correlation between IL-15 and IGF-1 is formed in wounded epidermis, and thereby amplifies IGF-1 production in epidermis for promoting re-epithelialization and wound healing. Meanwhile, keratinocytes could also interact with Vγ4 T cells, which infiltrate in epidermis to form an IL-17A-IL-1β/IL-23 feedback loop to augment local inflammation for efficient skin wound healing. In the wounds of diabetic mice, DETC-mediated IL-15-IGF-1 correlation and Vγ4 T cell-mediated IL-17A-IL-1β/IL-23 loop are coordinated to improve the defects of diabetic wound healing through enhancing re-epithelialization and local inflammation, respectively.

IL-2 and IL-15 participate in the survival and activation of γδ T cells (25, 49, 50). Both interact with α, β, or γ_c_ chains of the receptor complexes (50, 51). Although IL-15 is similar to IL-2 in its biological properties and three-dimensional configuration, IL-15 is more important than IL-2 for the survival and proliferation of DETCs upon TCR engagement, because IL-15 and not IL-2 is expressed in the epidermis under a steady state (25). In addition, mature fetal Vγ5 thymocytes and DETCs express the IL-15Rβ chain (CD122) but not IL-2Rα (CD25) (11). Compared to wild-type controls, the number of mature Vγ5 T cells is reduced in the fetal thymus and DETCs are absent in IL-15^−/−^ mice, while the number of mature Vγ5 T cells is normal in the fetal thymus and DETCs survive in the adult skin of IL-2^−/−^ mice (25). Therefore, IL-15 rather than IL-2 seems to be necessary for DETC homeostasis in the skin (25, 35). Moreover, activated DETCs secrete IL-15 but fail to produce IL-2, indicating that IL-15 is more important than IL-2 for the efficient activation of DETCs at the early stages of wound healing. Liu et al. and Wang et al. have demonstrated that IL-15 rescues the insufficient activation of DETCs and increases IGF-1 production by DETCs, and IGF-1 in turn induces keratinocytes to secrete IL-15 in diabetic mice (26, 28). Their work indicates that IL-15 and IGF-1 are positively correlated in the wounded epidermis to promote re-epithelialization (Figure 1). Furthermore, the regulation of the IGF-1-IL-15 loop partially depends on the mTOR pathway (26–28).

## Activated DETCs Promote Re-Epithelialization by Producing IGF-1 and KGF-1/2

IGF-1 is primarily produced in the liver, but it is also derived from DETCs in the epidermis. DETCs constitutively generate IGF-1, and keratinocytes express IGF-1R under normal conditions (4). IGF-1 combined with IGF-1R can trigger phosphoinositide 3-kinase and mitogen-activated protein kinase pathways to protect keratinocytes from apoptosis and differentiation (4, 52, 53). Beyond secreting a small amount of IGF-1 in the steady state, DETCs also express IGF-1R to maintain survival in the epidermis *via* an autocrine pathway (4). Phosphorylated IGF-1R is increased at wound margins 24 h after injury, and upregulated IGF-1 protects keratinocytes from apoptosis in damaged areas to assist re-epithelialization (4).

Dendritic epidermal T cells do not secrete KGFs (KGF-1 and KGF-2) in homeostasis conditions, but rapidly produce KGFs upon wounding (3). Keratinocytes constitutively express KGF receptor FGFR2-IIIb, and thus KGFs derived from DETCs can bind FGFR2-IIIb receptor to induce the proliferation and migration of keratinocytes during the re-epithelial phase of wound healing (3, 54). FGFR2-IIIb is not expressed on DETCs, showing that KGFs do not reversely regulate the effector functions of DETCs under stressed conditions (3). DETCs can also secrete TGF-β to aid tissue repair; release GM-CSF XCL1, CCL3, CCL4, CCL5, and hyaluronan to recruit leukocytes to wound sites; and produce IL-17, IFN-γ, and TNF-α to facilitate inflammation (55, 56).

## The Development of Vγ4 T Cells in the Thymus

Vγ4 TCR is rearranged in the late fetal thymus from ED 17 until birth and afterward (57, 58). Vγ4 T cells develop into two main subsets: IL-17A^+^Vγ4 T cells with the phenotype of CCR6^+^CD27^−^, and IFN-γ^+^Vγ4 T cells with CCR6^−^CD27^+^ (59). Certain embryonic thymus conditions are required for γδ T cells to acquire the capacity to produce IL-17A. IL-7 is necessary for the development of γδ T17 cells in the thymus, which can promote the accessibility of the TCR γ locus to V(D)J recombinase and regulate the differentiation of γδ T cells preferentially toward the CD27^−^IL-17A^+^ subset (15, 60). CCR6^+^CD27^−^γδ T17 cells express the subunit of IL-17A/F receptor IL-17RC, which is not detected on CCR6^−^CD27^+^γδ T cells (61). In the absence of IL-17A, CCR6^+^CD27^−^γδ T17 cells become overabundant in the thymus and secondary lymphoid organs, indicating that the development and homeostasis of γδ T17 cells is restricted by IL-17A in a negative feedback loop (61). Moreover, transcription factor Sox13 is required for the maturation of IL-17A^+^Vγ4 T cells in the neonatal thymus, and its mutation is able to protect mice from psoriasis-like dermatitis (62).

## Vγ4 T Cells are the Dominant Subset of Murine Dermal γδ T Cells

When exiting the thymus, Vγ4 T cells have obtained stem cell-like properties of self-renewal and are radiation resistant (63). Vγ4 T cells are localized to the secondary lymphoid organs as the dominant subset of murine peripheral γδ T cells, and they are also distributed in the dermal layer of murine skin (63). Vγ4 T cells comprise nearly 50% of dermal γδ T cells, though Vγ1, Vγ5, Vγ6, and Vγ7 T cells also exist in the dermis (64). Vγ4 T cells, as the major γδ T cells in the dermis, are capable of secreting IL-17A and IFN-γ, which play distinctive roles in autoimmune diseases, graft rejection, antiviral immunity, and antitumor responses (6, 10, 33, 65).

## Vγ4 T Cells Provide the Major Source of IL-17A at the Early Stage of Skin Inflammation

Vγ4 T cells have been reported to participate in autoimmune diseases and skin graft rejection at the early stages by producing IL-17A (10, 33, 62, 66). IFN-γ-positive Vγ4 T cells play a protective role in antitumor immunity, but they do not contribute in skin transplantation and wound healing (10, 33, 67). Which cytokine Vγ4 T cells secrete may depend on local circumstances. As it is well-known that Th17 cells are a major source of IL-17A in the adaptive immune response, Vγ4 T cells act as an innate source of IL-17A before Th17 cells play their roles (68). Vγ4 T cells have some features in common with Th17 cells, such as IL-23 receptor, CCR6, and RORγ (68). However, Vγ4 T cells have gained the potent ability to produce IL-17A and express dectin-1 and TLRs when they egress from the thymus and, therefore, they can directly interact with pathogens and secrete IL-17A as the first line of defense against bacterial pathogens (61, 68). Vγ4 T cells also produce IL-17A to induce psoriasis-like skin inflammation, and IL-17A-positive T cells expand promptly in draining lymph nodes when exposed to the inflammatory agent imiquimod (64, 69). Furthermore, we have reported recently that Vγ4 T cells provide a major source of IL-17A in the epidermis at the early stages of wounding. Approximately half of the epidermal IL-17A-positive cells are Vγ4 T cells after skin injury (67). IL-17A production in the epidermis is dramatically decreased after the depletion of Vγ4 T cells in wild-type mice, but it is significantly enhanced in *Tcr*δ^−^*^/^*^−^ animals by the addition of freshly isolated Vγ4 T cells onto wound beds (67). In addition, Vγ4 T cells also migrate to noninflamed skin and peripheral lymph nodes, and respond faster and stronger to a second imiquimod challenge (69). Expanded Vγ4 T cells in lymph nodes can infiltrate back into inflammatory skin *via* S1P1 with similar migratory mechanisms as conventional αβ T cells (70). Of note, we purchased anti-Vγ4 TCR (UC3-10A6) antibody (Ab) from BioXcell to deplete Vγ4 T cells according to previous research (71, 72). However, *in vivo* treatment with both GL3 and UC7-13D5 antibodies against TCR, as identified by Koenecke et al., caused TCR internalization instead of γδ T cell depletion (73). Therefore, we cannot exclude the possibility that Ab treatment cannot eliminate γδ T cells, but instead decreases TCR complexes on the cellular surface.

## Vγ4 T Cell-Derived IL-17A and Epidermal IL-1β/IL-23 Form a Positive Feedback Loop to Amplify Local Inflammation after Skin Injury

**Figure 2:**
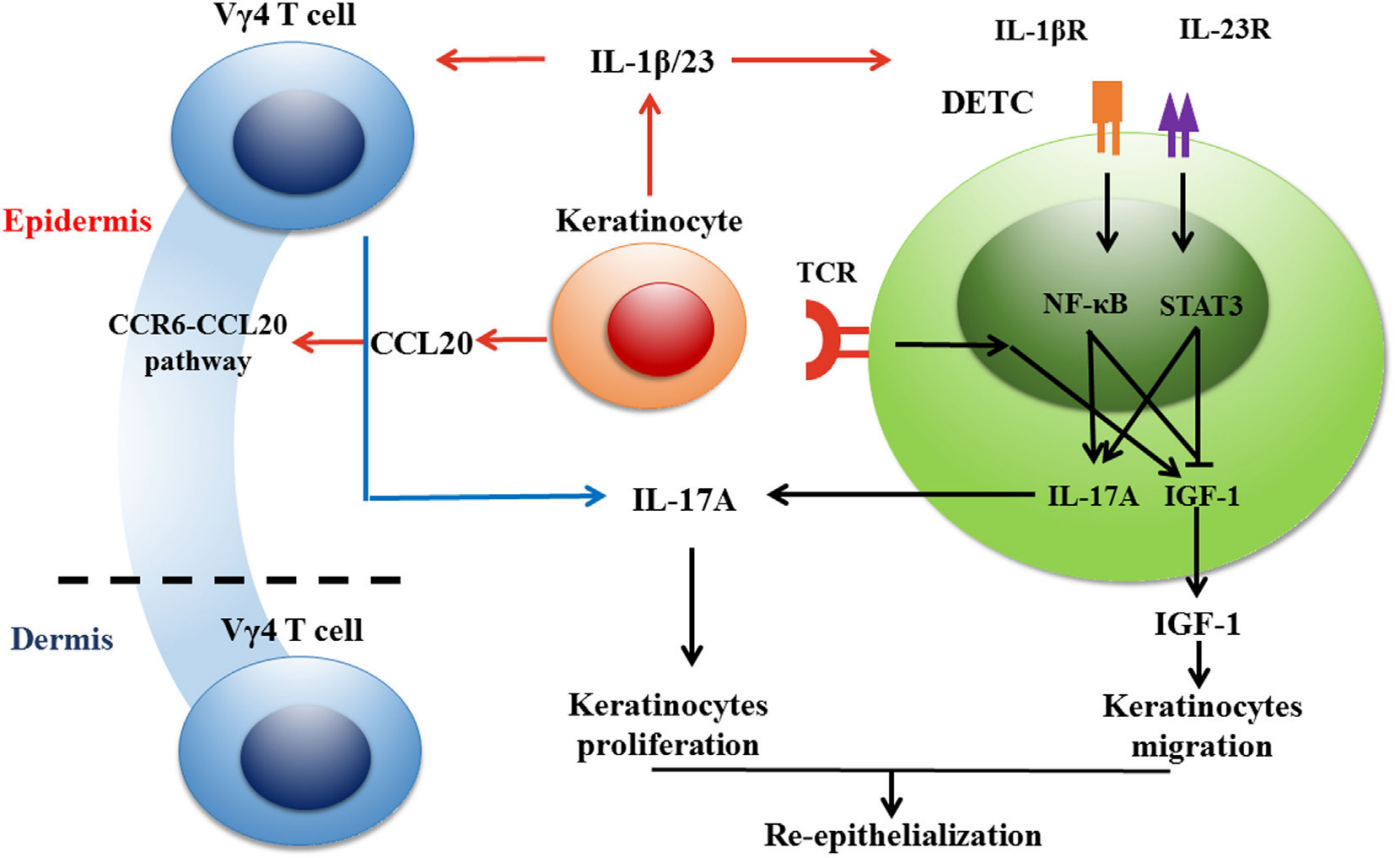
Vγ4 T cells inhibit the production of IGF-1 in dendritic epidermal T cells (DETCs) *via* IL-17A-IL-1β/IL-23 loop and thereby delay wound healing in normal mice. Once keratinocytes get stressed or damaged, DETCs are activated quickly through TCRs by sensing some as-yet-unknown antigens expressed by damaged keratinocytes in a non-major histocompatibility complex restricted manner. Dermal Vγ4 T cells are attracted to the epidermis around wounds by CCL20, which is increased in wounded epidermis, and provide an early source of IL-17A to delay skin wound healing. IL-17 secreted by Vγ4 T cells inhibits IGF-1 production by DETCs in an indirective way where IL-1β and IL-23 produced by keratinocytes act as the bridge between them. IL-1β and IL-23 are key cytokines to suppress IGF-1 production by DETCs. Besides, Vγ4 T cell-derived IL-17A facilitates keratinocytes to secrete IL-1β and IL-23, and thus forms a positive feedback with keratinocyte-derived IL-1β and IL-23. It is worthy to note that IL-1β is more effective than IL-23 in our investigation. NF-κB pathway plays a crucial role in IL-1β-suppressed IGF-1 expression in DETCs. In conclusion, for wounds in normal mice, Vγ4 T cell-mediated IL-17A-IL-1β/IL-23 loop has a negative impact on IGF-1 production by DETCs and thereby delays skin wound closure.

The IL-1β/IL-23-IL-17A axis is critical for the initiation and amplification of inflammatory responses (5, 6, 69, 74, 75). IL-17A has been demonstrated to act upstream to enhance epidermal IL-1β/IL-23 production in a skin graft transplantation model (10). Furthermore, IL-1β and IL-23 production in the epidermis of wound edges is weakened by a deficiency or blockage of IL-17A but enhanced by the addition of rIL-17A (67). Depletion of Vγ4 T cells reduces epidermal IL-1β/IL-23 production, but supplementing wild-type rather than *Il-17a*^−^*^/^*^−^ Vγ4 T cells onto wound beds promotes epidermal IL-1β/IL-23 production in *Tcr*δ^−^*^/^*^−^ mice (67). Therefore, we regard that IL-17A secreted by Vγ4 T cells and IL-1β/IL-23 derived from epidermal cells may form a positive feedback loop in the epidermis around wounds to amplify local inflammation after skin injury. In addition, IL-17A-producing γδ T cells express high levels of CCR6 on their surface and are recruited by CCL20 to inflammatory sites (76). CCL20 neutralization dramatically decreases the infiltration of Vγ4 T cells into the epidermis around wounds and reduces epidermal IL-17A production. Together, IL-1β/IL-23 and CCL20 have the ability to amplify IL-17A production by Vγ4 T cells and thereby exacerbate local inflammation in the epidermis after skin injury (Figure 2). However, the capability of Vγ4 T cells to produce IL-17A can be repressed by B and T lymphocyte attenuator, which decreases the accumulation of Vγ4 T cells in imiquimod-induced inflammatory skin and draining lymph nodes (77). Whether IL-17A production by Vγ4 T cells is negatively regulated by BLTA in wound healing needs further investigation.

## IL-17A is Required for Efficient Skin Wound Healing, but Excessive IL-17A Retards Wound Repair

IL-17A is an important pro-inflammatory cytokine that plays a critical role in the initiation and amplification of inflammation responses. IL-17A is required for efficient skin wound healing. *Il-17a*^−^*^/^*^−^ mice exhibit defects in wound repair, which can be restored by the addition of rIL-17A and IL-17A-producing DETCs (9). Moreover, IL-17A production is reduced in skin around wounds of diabetic mice, and IL-17A-positive Vγ4 T cells transferred to the wound bed can improve wound healing (78). In addition, DETCs provide a source of epidermal IL-17A after skin injury, which accelerates wound healing by inducing epidermal keratinocytes to express the host-defense molecules β-defensin 3 and RegIIIγ (9). However, Rodero et al. reported a contradictory role for IL-17A in skin wound repair and found that the application of an IL-17A-neutralizing Ab onto the wound bed significantly promoted wound healing (8). To reconcile these conflicting roles of IL-17A in skin wound healing, Li et al. blocked IL-17A with an overdose of neutralizing Ab (200 μg/wound) in wound margins, which led to defective skin wound closure, indicating that IL-17A is essential for efficient wound healing. However, the addition of a moderate dose of anti-IL-17A neutralizing Ab (20 μg/wound) significantly improved skin wound repair. In addition, a high dose of rIL-17A (200 ng/wound) rather than low or medium doses (2 or 20 ng/wound) injected into the wound bed prominently delayed skin wound healing, suggesting that excessive IL-17A has a negative impact on skin wound repair (67). These facts strongly suggest that IL-17A plays dual roles: moderate IL-17A is required for efficient skin wound healing, but excessive IL-17A dampens skin wound closure. We consider that these dual roles do not coexist at the same time, but rather depend on the concentration of IL-17A under the circumstances. Mild amounts of IL-17A at the wound edge re-establish the antimicrobial skin barrier after skin injury by inducing epidermal keratinocytes to express antimicrobial peptides and proteins, but superfluous IL-17A induces the IL-1β/IL-23-IL-17A loop to amplify local inflammation, thus inhibiting wound repair.

## Vγ4 T Cells Inhibit IGF-1 Production of DETCs to Delay Skin Wound Closure through IL-17A

Vγ4 T cells secrete IL-17A to delay wound repair, but IL-17A fails to directly affect the pro-healing function of DETCs, as IGF-1 expression by DETCs is not able to be directly reduced by IL-17A (67). However, epidermal IL-1β and IL-23 are key factors for the suppression of IGF-1 in DETCs. Taken together with the positive loop of IL-17A and IL-1β/IL-23, it is very likely that IL-1β and IL-23 act as the bridge between Vγ4 T cells and DETCs. Furthermore, IL-1β and IL-23 notably promote the phosphorylation of NF-κB and STAT3 and facilitate their translocation from the cytoplasm to the nucleus in DETCs. However, IL-1β shows more significant effects on DETCs than IL-23, which only exhibits synergic inhibition with IL-1β (67). Therefore, we consider that IL-17A produced by Vγ4 T cells indirectly impedes DETCs to secrete IGF-1 and delays skin wound closure with mediators IL-1β and IL-23 (Figure 2).

## A Potential Functional Link Between Vγ4 T Cells, Epidermal Cells, and DETCs in Skin Wound Healing

Upon skin injury, epidermal cells interact with DETCs to form an IL-15-IGF-1 loop to amplify IGF-1 production in the epidermis for re-epithelialization (26, 28). Meanwhile, epidermal cells can also interact with epidermis-infiltrating Vγ4 T cells to form an IL-17A-IL-1β/IL-23 loop to augment local inflammation (78). In diabetic wounds, the DETC-mediated IL-15-IGF-1 loop and Vγ4 T cell-mediated IL-17A-IL-1β/IL-23 loop improve the defects of diabetic wound healing by enhancing re-epithelialization and local inflammation, respectively (Figure 1). However, in normal wounds, the Vγ4 T cell-mediated IL-17A-IL-1β/IL-23 loop has a negative impact on IGF-1 production by DETCs and thereby delays skin wound closure (Figure 2). This indicates that a balance exists between Vγ4 T cell-derived IL-17A and DETC-derived IGF-1 for optimal skin wound healing.

Some interesting issues need to be further investigated in the near future: the precise underlying mechanisms of IL-1β and IL-23 inhibition of IGF-1 production in DETCs, and the influence of co-stimulatory molecules on the two loops during wound healing. Whether epidermal stem cells are involved in the regulation of IL-17A and IGF-1 during re-epithelialization, and how IL-17A and IGF-1 play roles in the homeostasis, migration, proliferation, and differentiation of epidermal stem cells still remains unknown.

## Author Contributions

Writing the original draft: YL and WH. Writing reviews and editing: WH, GL, and JW. Funding acquisition: WH and GL. Supervision: GL and WH.

## Conflict of Interest Statement

The authors declare that the research was conducted in the absence of any commercial or financial relationships that could be construed as a potential conflict of interest.
